# Single cell correlation analysis of liquid and solid biopsies in metastatic colorectal cancer

**DOI:** 10.18632/oncotarget.27271

**Published:** 2019-12-17

**Authors:** Anna S. Gerdtsson, Jana-Aletta Thiele, Stephanie N. Shishido, Serena Zheng, Randolph Schaffer, Kelly Bethel, Steven Curley, Heinz-Josef Lenz, Diana L. Hanna, Jorge Nieva, Anand Kolatkar, Carmen Ruiz, Mariam Rodriguez-Lee, Gerard J. Oakley III, Jerry S.H. Lee, James Hicks, Peter Kuhn

**Affiliations:** ^1^USC Michelson Center for Convergent Bioscience, University of Southern California, Los Angeles, CA, USA; ^2^Biomedical Center, Faculty of Medicine in Pilsen, Charles University, Pilsen, Czech Republic; ^3^Scripps MDAnderson Cancer Center, La Jolla, CA, USA; ^4^Division of Surgical Oncology, Baylor College of Medicine, Houston, TX, USA; ^5^Keck School of Medicine, University of Southern California, Los Angeles, CA, USA; ^6^Diagnostic and Experimental Pathology, Eli Lilly and Company, Lilly Corporate Center, Indianapolis, IN, USA

**Keywords:** single cell analysis, liquid biopsy, metastatic colorectal cancer, circulating tumor cells, cytomorphology

## Abstract

As cancer care is transitioning to personalized therapies with necessary complementary or companion biomarkers there is significant interest in determining to what extent non-invasive liquid biopsies reflect the gold standard solid biopsy. We have established an approach for measuring patient-specific circulating and solid cell concordance by introducing tumor touch preparations to the High-Definition Single Cell Analysis workflow for high-resolution cytomorphometric characterization of metastatic colorectal cancer (mCRC). Subgroups of cells based on size, shape and protein expression were identified in both liquid and solid biopsies, which overall displayed high inter- and intra- patient pleomorphism at the single-cell level of analysis. Concordance of liquid and solid biopsies was patient-dependent and between 0.1-0.9. Morphometric variables displayed particularly high correlation, suggesting that circulating cells do not represent distinct subpopulations from the solid tumor. This was further substantiated by significant decrease in concentration of circulating cells after mCRC resection. Combined with the association of circulating cells with tumor burden and necrosis of hepatic lesions, our overall findings demonstrate that liquid biopsy cells can be informative biomarkers in the mCRC setting. Patient-specific level of concordance can readily be measured to establish the utility of circulating cells as biomarkers and define biosignatures for liquid biopsy assays.

## INTRODUCTION

Colorectal cancer (CRC) is the third most common cancer globally [1]. The liver is the dominant metastatic site for patients with CRC due to hepatic portal circulation drainage from the colon. According to the spreader/sponge model [2], the liver is also a critical metastatic spreader of CRC [3]. Isolated liver metastases from CRC are routinely resected with curative intent, but despite metastasectomy being associated with long-term relapse-free survival, approximately one third of patients relapse within 2 years after hepatic resection [4], suggesting the potential role of distant micrometastasis or circulating tumor cells (CTCs). In the current study, blood and tumor touch preparations (touch preps) were collected from patients undergoing hepatic resection of metastatic CRC (mCRC) with the purpose of determining the morphometric concordance of liquid and solid biopsy cells in this disease setting.

With the increasing range of approved drugs and the recognition that targets may bridge across different organ sites, the need for companion and complementary diagnostic biomarkers is becoming a more frequent requirement. Development of these diagnostics within specific clinical contexts requires an understanding of underlying mechanisms to decipher the complexity of tumor evolution. While many current biomarkers are tissue-based, requiring a solid biopsy with limited sampling frequency, there is significant interest in determining whether minimally invasive liquid biopsies that utilize measurements of factors that exist in the blood, such as CTCs, can reflect the state of solid tumors to complement, or substitute, solid biopsies. To faithfully capture cell heterogeneity, personalized oncology is furthermore moving towards single cell profiling for more informed clinical decision making, as opposed to homogenized cell populations in conventional bulk biopsy analysis [5]. To this end, there is a need for quantitative and reproducible single cell analysis strategies, preferably with the potential of multi-omics profiling to measure essential markers for cell identity and function. In comparison to single analyte bulk liquid biomarkers, e.g. circulating tumor DNA (ctDNA) or serum proteins, CTCs can provide tissue-based information on genomic, proteomic and morphometric profiles of the tumor, thus representing a complementary source for less invasive, high-definition tumor tissue characterization. To date, few studies have been conducted with the intention of investigating the level of correlation of circulating cells to the solid biopsy; particularly, concordance of cytomorphology has not yet been well characterized or quantified.

High-Definition Single Cell Analysis (HD-SCA) is a workflow for detailed characterization of circulating rare cells by high resolution imagery of individual cells. By screening all nucleated cells within a sample, it is a ‘no cell left behind’ approach enabling both increased sensitivity compared to most other CTC assays, as well as inherent morphometric profiling of all candidate tumor cells. Importantly, downstream analyses are designed for a standard slide format and include genomic profiling of individual cells by isolation and sequencing [6, 7], or multiplexed protein profiling by imaging mass cytometry [8], enabling molecular characterization in addition to enumeration and morphometric analysis. The analytical validation of the HD-SCA workflow has been completed for CTCs in a range of cancers [9–11], and predictive liquid biopsy tests based on the technology are available through a CLIA/CAP certified laboratory [12]. With an earlier version of the workflow, a case study of CTCs and primary cells from lung cancer concluded that a subset of CTCs displayed similar cytomorphology to a subset of primary tumor cells, even though the solid biopsy had been collected years prior to the CTCs [13]. Applying the same technology, a cytomorphologic visual evaluation of CTCs in a small case series of CRC patients indicated that CRC CTCs retain the morphology of the solid tumor [14]. Based on the current HD-SCA workflow, which includes a more detailed digital analysis of candidate cells and allows for incorporating solid biopsy single cell analysis, we set out to establish an approach for quantitative correlation analysis of liquid and solid biopsy cells based on high-content morphometric analysis on the single-cell level.

## RESULTS

### Liquid and solid biopsy HD-SCA workflow harmonization and validation for CRC analysis

**Figure 1:**
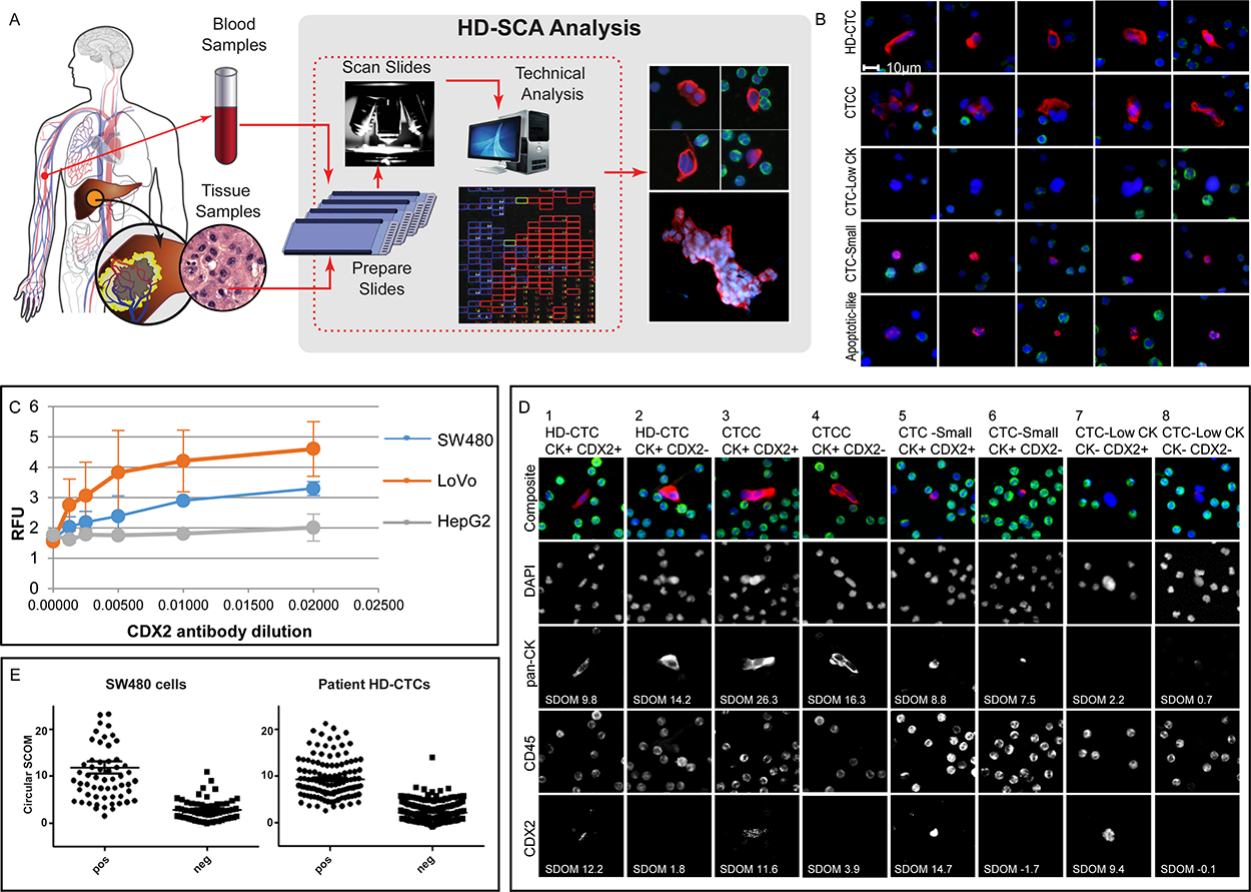
HD-SCA sample processing and CRC analysis overview. **(A)** Peripheral blood (liquid biopsies) collected prior to surgery and tumor touch preps (solid biopsies) collected from each piece of resected liver (or colon) tumor were processed, stained, and imaged in parallel. Candidate tumor cells were re-imaged at higher resolution and segmented for collection of single cell imaging data. **(B)** Cells of different categories as classified in the HD-SCA report system identified in mCRC patients. Each row of composite images represents one cell category (DAPI, blue; pan-CK, red; CD45, green). HD-CTCs and CTCCs were included in the analysis. Other cell categories classified in the HD-SCA report system are CTC-Low CK (CK-/low, CD45- morphologically distinct and larger than surrounding WBCs), CTC-Small (CK+, CD45- with similar size as surrounding WBCs), Apoptotic-like (CK+, CD45- cells with irregular or disrupted nucleus and/or cytoplasm) as well as False Positives and WBCs (not shown). **(C)** A CDX2 monoclonal antibody was selected after an initial antibody screening using plated positive (SW480 and LoVo) and negative (HepG2) control cell lines and titrated in 3 independent experiments (RFU=Relative Fluorescence Units). **(D)** SW480 cells were spiked in whole blood and processed according to the HD-SCA protocol. The CDX2 SDOM of tumor cells relative to surrounding WBCs was similarly distributed for CDX2+ and CDX2- cells in spiked SW480 cells and patient HD-CTCs from the first HD-CTC positive patient (CRC004). **(E)** Examples of cell subgroups as defined by HD-SCA cell category and CK and CDX2 expression, identified from a single sample from patient CRC112. Images were taken at 40X magnification, except for cell 7 where images were taken directly from the 10X scanning report due to lack of 40X imagery. Each column depicts the target cell(s) with a composite image (DAPI, blue; pan-CK, red; CD45, green) on top and the individual channels below.

The primary objective of the study was to explore the relationship of liquid and solid cell morphology and to develop an approach for assessing the concordance of liquid biopsy cells to the solid tumor. The tumor touch prep format was used as representative solid biopsy samples as it could be readily integrated in the existing HD-SCA platform originally developed for high-resolution characterization of CTCs. Unlike FFPE samples, the HD-SCA solid and liquid biopsy processing provides fresh and intact cells enabling a comparable downstream single whole cell characterization. The harmonized workflow is outlined in Figure 1A. Candidate cells identified by the HD-SCA algorithm were pre-classified into different cell categories (Figure 1B). The HD-CTC category of CK+, CD45-, morphologically distinct cells with a nuclear size larger than surrounding WBCs, were subdivided into single cells and CTC Clusters (CTCC) of two or more HD-CTCs. All predefined HD-SCA cell categories were identified within the mCRC cohort. All cell categories were enumerated, and each cell annotated with 10X imaging data stored in a database. Cells of the HD-CTC and CTCC categories were included in the subsequent comparison of liquid and solid cells.

**Figure 2:**
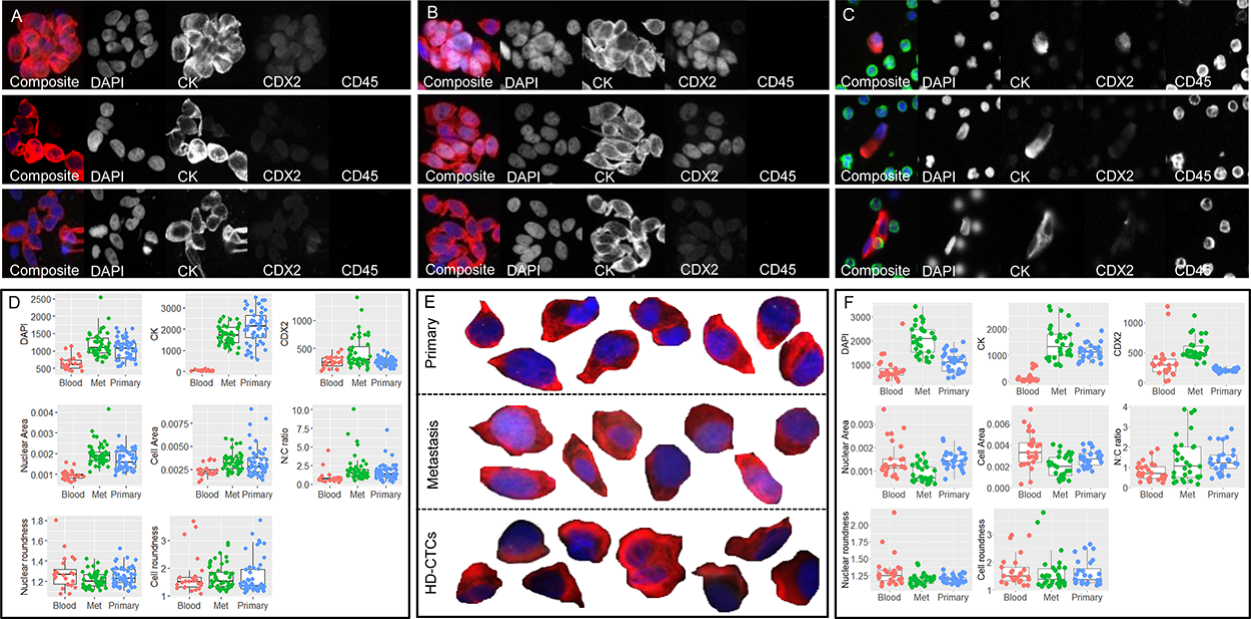
Primary, metastatic and HD-CTC comparison for patients CRC703 **(A**–**D)** and CRC009 **(E**–**F)**. Tumor touch preps from (A) primary CRC tumor and (B) hepatic metatasis. (C) Liquid biopsy HD-CTCs. In A-C, each row shows one cell or cell cluster with the composite image followed by the 4 individual channels. (D) Selected raw measurements on all segmented single cells from patient CRC703. (E) Selected (enlarged) segmented composite images of primary CRC cells (top row), metastasis cells (middle row) and circulating HD-CTCs (bottom row). (F) Selected raw measurements on all segmented single cells from patient CRC009. The boxplots show Blood, HD-CTCs (red); Met, metastatic cells (green); Primary, primary CRC cells (blue). Measurements include mean pixel intensity (RFU) for DAPI, CK and CDX2, each measured using the same exposure across sample types for each patient, nuclear area and cell area (mm2), nuclear-to-cytoplasm (N/C) ratio, nuclear roundness and cell roundness (perimeter^2/(4*pi*area). Composite images show DAPI (blue), CK (red), CDX2 (white) and CD45 (green).

An assay was developed specifically for characterization of CRC using primary (SW480) and metastatic (LoVo) CRC cells and negative hepatocellular carcinoma (HepG2) cells spiked in normal donor whole blood and processed according to standard HD-SCA procedure. CDX2 expression was higher in metastatic than in primary CRC cells, and absent in hepatocellular carcinoma (Figure 1C). Following antibody screening and optimization, the final assay showed a slide-to-slide coefficient of variation of 0.15 for identification of spiked cells (114±17 cells/slide on n=7 slides with spiking rate aimed at 100 cells/slide). The model cells displayed a heterogeneous CDX2 expression, with 50% of spiked SW480 cells, prepared, processed, stained and imaged in the same batch, classified as CDX2-positive, while HD-CTC-positive clinical samples had a positivity rate of 39%. Based on the background of surrounding WBCs, the CDX2 signal had an average Standard Deviation Over the Mean (SDOM) of 11.8 and 9.4, for CDX2 positive spiked cells and patient cells, respectively. (Figure 1D). The threshold for CDX2 positivity was indicated at SDOM 5 for both model cells and patient HD-CTCs but was not applied as a strict cut-off value for CDX2 classification. The CDX2 classification was rather determined on a cell-by-cell basis due to that an imperfect segmentation resulted in that a few clearly CDX2-positive cells had a SDOM below 5 (Figure 1E). In addition, SDOM values could not be robustly computed for touch prep samples which frequently lacked WBCs for accurate determination of background level by this means. Thus, based on visual evaluation and using SDOM values as guidance, candidate cells classified into the predefined HD-SCA categories were further subdivided into CDX2+/- bins. Both CDX2+ and CDX2- cells were observed within each cell category, across and within patients, demonstrating the intra-sample heterogeneity of rare circulating cells, even within single slides (Figure 1E). Of note, the touch preps also displayed highly heterogeneous CDX2 expression, with cells ranging from CDX2- to strongly CDX2+, sometimes even within the same cell cluster (Figure 2A, 2B).

### Single-cell morphometric characterization of liquid and solid biopsies

In total, pre-surgery blood and one or more solid biopsies were collected from 43 mCRC patients undergoing hepatic metastasectomy with curative intent (Table 1). Fifteen patients (35%) had HD-CTC positive pre-surgery samples (>4 HD-CTCs/mL). The relatively low incidence of HD-CTC positive patients could possibly be explained by the pre-selection of patients with tumors deemed surgically resectable for curative intent, thus less advanced than the majority of patients diagnosed with stage IV mCRC. The majority of patients had also been treated with neoadjuvant drugs and/or chemoradiation prior to surgery. Of the 15 HD-CTC positive patients, 5 were excluded by criteria set up by the power analysis (<20 HD-CTCs/2 slides or <4 monolayer clusters of intact cells, see methods section and Supplementary Table 1). Samples from the remaining 10 patients were included in the liquid and solid biopsy correlation analysis. For 3 of these patients, both primary and metastatic solid biopsies had been collected at the time of surgery. A total of 1058 CRC cells were segmented with an average of 43 cells per tissue type (HD-CTCs, hepatic metastasis and primary CRC) per patient.

**Table 1:** Clinicopathological characteristics

		Total HD-CTC positive*	HD-CTC negative
**Total**	43 (100%)	15 (35%)	28 (65%)
			
**Gender**			
Female	22 (51%)	8 (53%)	14 (50%)
Male	21 (49%)	7 (47%)	14 (%0%)
			
**Age at Consent**	57±12 (27-79)	53±13 (27-71)	60±10 (43-79)
(years±stdev (range))			
			
**Ethnicity**			
Asian	5 (12%)	1 (7%)	4 (14%)
Black or African American	5 (12%)		5 (18%)
Hispanic or Latino	5 (12%)	1 (7%)	4 (14%)
White	26 (61%)	13 (87%)	13 (46%)
NA	2 (5%)	2 (7%)	
			
**Weeks from diagnosis to consent**	87±86 (2-364)	77±88 (2-334)	94±86 (18-364)
(weeks±stdev (range))			
			
**Primary tumor location**			
Cecum	1 (2%)	1(4%)	
Ascending	8 (19%)	3 (20%)	5 (18%)
Transverse	3 (7%)	2 (13%)	2 (7%)
Descending	2 (5%)	1 (7%)	1 (4%)
Sigmoid	9 (21%)	3 (20%)	6 (21%)
Rectum	11 (26%)	4 (27%)	8 (29%)
Sigmoid+Rectum	3 (7%)	2 (13%)	2 (7%)
NA	6 (14%)	3 (11%)	
			
**Staging of primary tumor**			
T1	2 (5%)	1 (7%)	1 (4%)
T2	2 (5%)	2 (7%)	
T3	22 (51%)	9 (60%)	14 (50%)
T4	4 (9%)	2 (13%)	2 (7%)
NA	13 (30%)	3 (20%)	7 (25%)
			
**Differentiation of primary tumor**			
Well	1 (2%)	1 (7%)	
Moderately to well	6 (14%)	7 (25%)	
Moderately	20 (47%)	7 (47%)	14 (50%)
Moderately to poor	2 (5%)	1 (7%)	1 (4%)
Poor	1 (2%)	1 (7%)	
NA	13 (30%)	5 (33%)	6 (21%)
			
**KRAS**			
Mutated	11 (26%)	5 (33%)	6 (21%)
Wild-type	12 (28%)	4 (27%)	8 (29%)
NA	20 (47%)	6 (40%)	14 (50%)
			
**Neoadjuvant chemotherapy**			
Yes	27 (63%)	10 (67%)	18 (64%)
No	7 (16%)	3 (20%)	4 (14%)
NA	9 (21%)	2 (13%)	6 (21%)

Number of patients per groups is shown, with percentage of total patients, HD-CTC positive patients, and HD-CTC negative patients, respectivley in parenthesis for each category (rounded numbers)

*(>4 HD-CTCs/mL (HD-CTC = (High-Definition Circulating Tumor Cell)

Despite the pleomorphism observed within the HD-CTC compartment, liquid and solid biopsy cells displayed an overall similar intra-patient morphology (Figure 2). Based on raw data, solid biopsy cells had significantly higher pan-CK and DAPI signals than liquid biopsy cells in all patients, while CDX2 was significantly higher in metastatic cells compared to primary CRC and HD-CTCs in accordance with the observations in the model cell lines (Figure 1C). In comparison, the distribution of features based on size and shape was patient specific, as shown for 2 patients with both primary and metastatic solid biopsies (Figure 2D, 2F). For example, the nuclear and cellular areas were significantly smaller in liquid biopsy cells than metastatic or primary solid biopsy cells in patient CRC703 (Figure 2A–2D), while for patient CRC009, the metastatic cells tended to be smaller than primary cells and HD-CTCs (Figure 2E–2F). Notably, many of the cells in both the liquid and solid biopsy samples displayed a cell morphology characteristic of columnar cells, with an elongated shape, and with the nucleus frequently located at one end of the cell. Overall, the analysis demonstrated the feasibility of using touch preps as solid biopsies for analysis within the HD-SCA platform, and that touch prep cells could generally be robustly segmented and compared to liquid biopsy HD-CTCs.

**Figure 3:**
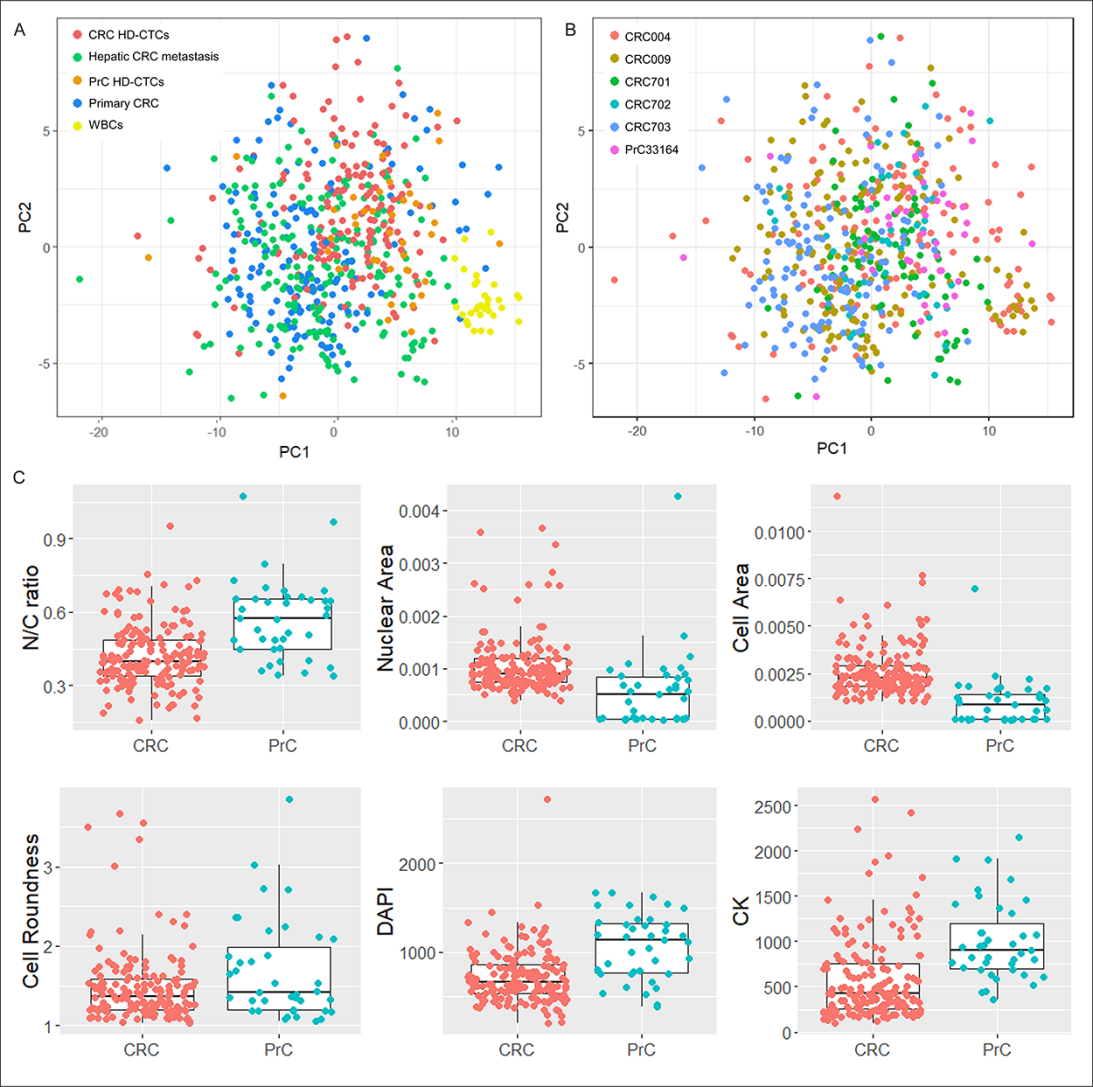
Distribution of CRC and PrC samples. **(A)** and **(B)** PCA of liquid and solid biopsies, including subsets of WBCs from HD-CTCs frames from CRC patients, and HD-CTCs from a PrC patient (USC33164). PC1 versus PC2 were plotted and samples colored according to tissue type **(A)** and patient **(B)**. **(C)** Boxplots of individual selected features displaying differential distributions in HD-CTCs from PrC and CRC.

Principal component analysis (PCA) was applied on combined data from CRC liquid and solid biopsy cells, WBCs segmented from the same images for a subset of cells, as well as HD-CTCs from a prostate cancer (PrC) patient that were imaged and segmented using the same method. Since the PrC sample had been stained with a different 4-color marker (androgen receptor), measurements based on that channel were excluded from the analysis. Based on the remaining features CRC liquid and solid cells and the PrC liquid cells clustered together and separate from WBCs, even though overlap was observed, showing that CRC HD-CTCs more resembled solid (primary and metastatic) CRC cells and genomically and morphologically well characterized HD-CTCs from a PrC patient [7] than surrounding WBCs from the same CRC patients (Figure 3A). Interestingly, the patients were not distinctly discriminated, indicating that cells were relatively similar also across patients based on unfiltered morphometric data (Figure 3B). Still, individual features were identified displaying significantly different distributions in the two cancers (Figure 3C). In particular, the overall cell size (p=5.3E-10) but also the nuclear size (p=4.0E-4) were significantly larger in CRC than in PrC, while the N/C-ratio was significantly larger in the PrC cells (p=1.9E-6). The PrC cells were also moderately rounder than the CRC cells (p=0.06) which, as demonstrated above, generally displayed a more elongated shape. The mean DAPI and CK signals were significantly higher in PrC than CRC (p=1.6E-6 and p=1.4E-6, respectively), although raw intensity values may not be reliably compared since the PrC and CRC samples were stained in different batches using different 4-color protocols. The full set of measured features on CRC samples were used to assess the influence of clinical parameters on the morphometric analysis (Supplementary Figure 1). Although subgroups based on clinical parameters within the 10 patients were small, analysis including all segmented CRC cells showed no significant impact of any of the clinical parameters collected. All 10 patients were of white ethnicity and had received neoadjuvant therapy, thus these 2 parameters could not be evaluated.

The computed features could be loosely grouped by type of measurement, i.e. object size, shape or biomarker distribution. Spearman correlation coefficients were computed based on the entire dataset, and features were ordered by hierarchical clustering (Supplementary Figure 2). As expected, the analysis showed that several features were highly correlated, implying that if desired, the number of computed measurements could potentially be refined to a more condensed morphometric signature for assessing cell relatedness, while preserving most of the information in the original data.

### Patient-specific liquid and solid biopsy concordance

**Figure 4:**
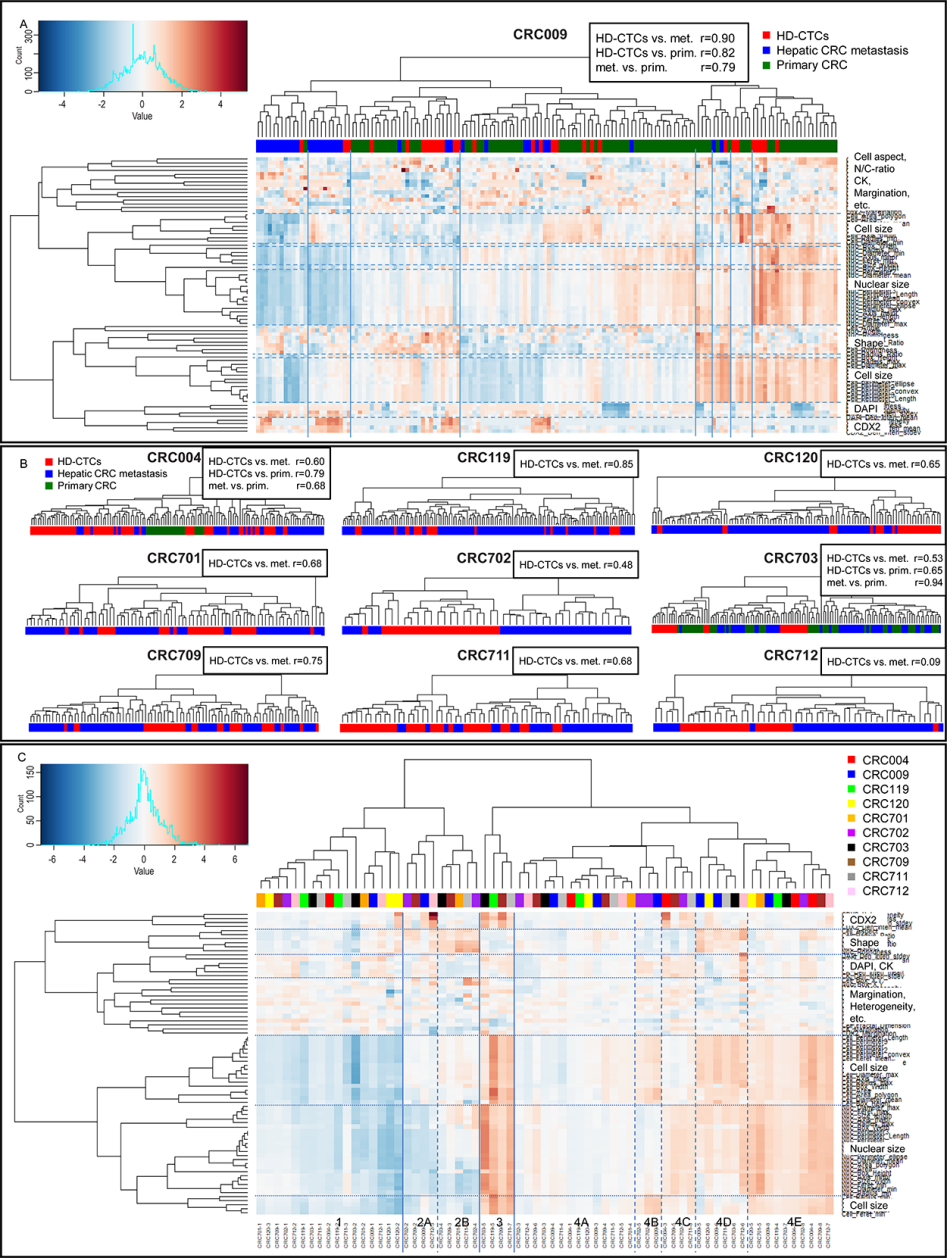
Hierarchical clustering and concordance analysis of liquid and solid biopsy cells. **(A)** Clustering and heatmap for patient CRC009 with 8 clusters defined by 3^rd^ level branching. The color bar represents tissue type. Pearson r correlation coefficients based on profiles of average scaled values of all features is displayed next to the heading. **(B)** Dendrograms with color bars and correlation coefficients for the other 9 patients. **(C)** Metacluster heatmap based on average values of patient-specific clusters. Four main clusters defined by 2^nd^ level branching with subclusters, are shown. The color bar represents the 10 patients. The main descriptor(s) of each feature cluster is shown on top of the individual feature annotations in A and C.

The liquid and solid biopsy concordance was assessed using hierarchical clustering of features normalized within each patient (Figure 4). For patient CRC009, 8 main clusters were observed (Figure 4A). Cluster 1 and 2 contained mainly metastatic cells, primarily characterized by small cell and nuclear size (cluster 1) and small nuclear size (cluster 2). Primary cells and HD-CTCs were distributed across most remaining clusters. The concordance of cells was further quantified through correlation coefficients of the profiles of average scaled values of all computed features. For CRC009, HD-CTCs were more correlated to both metastatic (r=0.90) and primary (r=0.82) cells than the two solid biopsies were to each other (r=0.79). The clustering and quantitative correlation analysis was repeated for the other 9 patients (Figure 4B). For CRC004, the primary CRC cells largely clustered together, however the quantified correlation suggested that the HD-CTCs more closely resembled primary (r=0.79) than metastatic (r=0.60) cells, and primary cells were also more correlated to the HD-CTCs than the metastatic cells (r=0.68). For CRC703, most HD-CTCs clustered separate from the solid cells, i.e. the primary and metastatic cells more resembled each other than the HD-CTCs, which was reflected in the correlation coefficient (r=0.94). Although lower correlation coefficients than the other two patients, the HD-CTCs were closer to the primary (r=0.65) than the metastatic (r=0.53) cells in this patient, like for CRC004. For the remaining 7 patients, only hepatic metastases were resected, with varying degree of cluster separation of HD-CTCs and solid cells (Figure 4B). Of note, patient CRC712 had significantly lower correlation of liquid and solid cells (r=0.09) compared to the other patients, due in part by an in average larger nuclear area of the liquid biopsy cells. The patient had received no chemotherapy prior to surgery and had no reported necrosis of the resected metastatic lesions. The blood sample had however been collected one week prior to the surgery, while for all other patients, both liquid and solid samples were collected at the day of surgery. We have currently no explanation to how the time of sampling could have impacted the result for this particular patient. Overall, the solid and liquid biopsy cell correlation had an average value of 0.65. Intraclass Correlation Coefficients (ICC), i.e. correlation of grouped rather than paired data, was marginally higher for solid cells (ICC=0.13) than HD-CTCs only (ICC=0.10) and lowest when including all cells (ICC=0.08), showing that the intra-patient concordance of cells in relation to the other patients was higher for solid cells. The overall low ICC values again indicated the heterogeneity of both solid and liquid cells, with a general low tendency for cells from the same patient to be more similar than across patients.

To assess the intra- and inter- patient concordance of cells and to further describe subgroups of cells present in the cohort, metaclusters were defined by mean values of each cluster from the individual patients (Figure 4C). The 10 patients were evenly distributed, demonstrating that cells with similar features were found across patients. Four main “clusters of clusters” were observed, primarily separated by cell and nuclear size, with cluster 1 containing small cells, cluster 2 average sized cells, cluster 3 large cells and cluster 4 average to large cells. Subgroups based on CDX2 and CK intensities, cell shape and N/C-ratio could also be delineated within the size-separated clusters (Table 2). All metaclusters contained both liquid and solid cells, except cluster 3, which consisted of a small number (1% of all cells) of large, elongated and CDX2-high cells only present in solid biopsies. In contrast, cluster 2B with average to small, round, CK-low and CDX2-low cells contained 15% primary cells, 85% HD-CTCs, and no metastatic cells. The remaining clusters consisted of a distribution of liquid and solid cells. The cell features that differed the most between liquid and solid cells, as well as the number of features with significantly different distributions also varied from patient to patient (Supplementary Table 2). Features based on CK intensity and intra-cell pixel distribution, including heterogeneity, margination and clumpiness, had consistently higher values (p<0.01) in solid cells across all 10 patients. In general, the most similar features in liquid and solid cells were those based on cell shape. None of the clinicopathological elements that were provided were found to be associated with patients displaying high versus low liquid and solid cell concordance.

**Table 2:** Metaclusters defining subgroups of cells of characteristic morphology and biomarker (CDX2, CK, DAPI) fluorescent signal, sorted in decreasing order of percentage of total cells. Presence of the different cell types are noted for each patient (grey filled cells)

Metacluster	Characteristic features	CRC 004	CRC 009	CRC 119	CRC 120	CRC 701	CRC 702	CRC 703	CRC 709	CRC 711	CRC 712	Percentage of total cells
4A	Average size, roundness and biomarkers intensity and distribution	X	X	X	X	X	X	X	X	X	X	48.3
1	Small cells, small nuclei, average roundness and biomarkers	X		X	X	X	X	X	X	X	X	18.6
4E	Average to large cells and nuclei, average roundness and biomarkers	X	X	X	X	X	X	X	X		X	18.3
4D	Average to large cells, round		X		X			X		X	X	4.2
2B	Average to small cell and nuclear size, round, CK-low, CDX2-low					X	X	X	X	X		3.5
4C	High N/C-ratio, elongated, CK- and DAPI-high	X					X		X	X		2.6
2A	Average cell size, small nuclear size, low N/C-ratio						X		X		X	2.3
4B	Low N/C-ratio, elongated, CK- and DAPI-high		X				X					1.3
3	Large cells, large nuclei, elongated, CDX2-high, CK- and DAPI-high			X				X	X	X		1.0

### HD-CTC levels in pre- and post-surgery liquid biopsies

**Figure 5:**
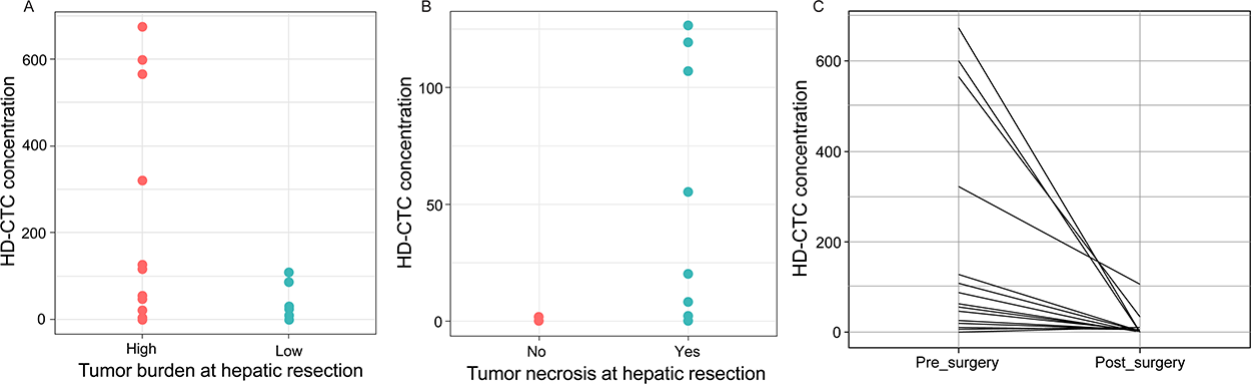
HD-CTC levels (cells/mL) in pre- and post-surgery liquid biopsies. **(A)** Patients with high tumor burden (synchronous disease or >4 hepatic metastases, n=20) had higher HD-CTC concentration than patients with low (<4 hepatic metastases, n=18) tumor burden at the time of hepatic resection, p=0.04. **(B)** Patients with necrotic metastases (n=9) had a higher HD-CTC concentration than patients with non-necrotic hepatic tumors (n=6), p=0.03. **(C)** HD-CTC concentration was significantly higher in pre-surgery than post-surgery samples (day-of-surgery), p=0.02.

Although the primary objective of the study on the HD-CTC positive subset of patients was the cytomorphometric comparison of solid and liquid cells, the association of HD-CTC concentration and clinical features was also evaluated using the full set of 43 patients. HD-CTC levels was significantly higher in patients with high tumor burden, i.e. primary CRC tumor and hepatic metastasis, or >4 hepatic tumor lesions present (Figure 5A). HD-CTC concentration was also significantly higher in patients with necrotic hepatic tumors than in patients with no apparent necrosis, implying that necrotic tumors have a higher propensity for shedding cells into the circulation (Figure 5B). Finally, the level of HD-CTCs decreased significantly from pre- to post-surgery sample, both collected at the day of surgery, again indicating that the circulating cells were rapidly cleared when the tumor was no longer present (Figure 5C). It should be noted that the observed decrease in HD-CTCs was not the cause of a dilution effect as the frequency of blood transfusions during surgery was negligible in the cohort. Thus, the level of HD-CTCs in the circulation was related to tumor burden and necrosis, implying that HD-CTCs could be informative biomarkers in mCRC.

## DISCUSSION

While solid tissue biopsy histology remains the gold standard for tumor diagnosis, the interest in using liquid biopsies for diagnostic and prognostic purposes is gaining more ground with the increasing demands of companion and complementary biomarkers for individualized treatments, for non-invasive longitudinal monitoring of disease evaluating response to approved agents, new drugs, or drug combinations [15]. Bulk analysis via ctDNA or solid tumor sequencing can still be used for specific molecular alterations. The obvious benefit of CTC analysis compared to bulk sequencing is however the capture of single cells, enabling characterization of tumor heterogeneity, temporal profiling of ratios of different clones present [7], and for the purpose of the current study, cytomorphometric comparison of solid and liquid biopsy tumor cells. While tests on ctDNA are currently making their way into the clinic [16–18], CTC assays have been slow to integrate into clinical care in general. Detection of CTCs through CellSearch® has been FDA approved for use in conjunction with clinical parameters as a prognostic biomarker in metastatic breast, prostate and colon cancer, but is rarely applied in clinical practice. Recently, a predictive test for resistance to targeted therapy in castrate resistant prostate cancer [12] has become available for patient care in the US as the first predictive test with proven clinical utility for this patient group, paving the way for clinical implementation of CTC testing.

One challenge for CTC assays has been the lack of studies showing correlation to the solid biopsy, which remains the gold standard for tumor characterization. The concordance of expression profiles of CTCs and solid tumor cells has previously been quantified using real time qPCR, but the studies have been hampered by low detection rate of genes in CTCs [19, 20]. In the current study, we set out to establish an approach to measure relatedness of liquid and solid cell cytomorphology in the mCRC setting. The tumor touch prep format was introduced to the HD-SCA workflow for morphometric liquid and solid cell concordance analysis using as few as 20 single cells per sample (Figure 1). We established an approach for liquid and solid single cell analysis based on high resolution imagery and nuclear and cytoplasmic segmentation and demonstrated the analytical performance of the HD-SCA technology for analysis of mCRC liquid and solid biopsies using a 4-color assay including CDX2. The rationale behind introducing CDX2 as a marker specific for CRC characterization was its potentially predictive role in CRC based on previously reported correlation between CDX2 expression and CRC grade, stage and metastasis [21, 22]. The application of the novel assay in the present cohort demonstrated that all previously defined HD-SCA cell categories were present in mCRC and displayed heterogeneous CDX2 and CK expression. Future and on-going studies include genomic and multiplexed proteomic analysis to further characterize the different cell categories (CDX2+ and CDX2-) in the mCRC setting and their relation to clinical outcomes.

Despite the observed pleomorphism in the liquid biopsy cell compartment, the distribution of several features was equivalent in HD-CTCs and solid cells. In general, features based on size and shape were more similar in the two, while measurements based on biomarkers showed larger variation (Figure 2, Supplementary Table 2). The observed variation in CK intensity and distribution could be due to biological differences between the cells, e.g., higher epithelial protein expression in solid cells than in circulating cells, which have previously been shown to have a more mesenchymal profile [23, 24]. The concordance of liquid and solid cells was however highly patient-specific and ranged between 0.1-0.9, with an average value of 0.65, based on morphometric profiles of average scaled values from single cells (Figure 4), and no clinicopathological elements were found to be associated with patients with low versus high liquid and solid biopsy cell concordance. The degree of liquid biopsy cell resemblance to primary versus metastatic cells varied for the 3 patients who presented with synchronous disease, with 2 of 3 patients showing a higher concordance of HD-CTCs to primary than to metastatic cells. Of note, resected colon tumors were in general significantly larger than the hepatic lesions, and may not have been as uniformly represented on the tumor touch preps, which could affect the variation in the level of pleomorphism and heterogeneity observed for the 3 patients from who primary colon tissue cells were analyzed. Despite levels of heterogeneity, the analysis across patients showed that liquid and solid biopsy mCRC cells were not readily discriminated from previously genomically well-characterized HD-CTCs from an unrelated PrC cancer patient, based on unsupervised clustering of the full set of measured features on single cells [7]. Univariate analysis still identified certain individual features that differed significantly between HD-CTCs from the two cancers. In particular, mCRC cells were larger than PrC cells, while PrC cells displayed a larger N/C-ratio and a rounder cell shape (Figure 3).

The traditional seed-soil theory has long overshadowed the possibility that blood biopsies might not fully reflect the solid tumor. More recently, it has been suggested that only specific subsets of tumor cells, e.g., cancer stem cells or cells undergoing epithelial to mesenchymal transition, have the survival benefits required to exist in circulation [25, 24]. Our results showed that although epithelial marker expression indeed was lower in the circulating cells, cell morphology, including the elongated cell shape characteristic of colon cells, was relatively concordant between liquid and solid cells. This may indicate that the bulk of circulating cells does not represent a distinct cell population from those of the solid tumor, at least not on a morphometrical level. Given the target population of patients undergoing neoadjuvant therapy prior to curative intent surgery, the rate of HD-CTC patients was relatively low as expected. The levels of HD-CTCs observed in positive samples in this study were however in many cases unusually high for CTC analysis. HD-CTC positivity was also associated with the presence of a tumor, as the cells were rapidly cleared once the tumor(s) had been resected, and the concentration was moreover significantly higher in patients with a higher tumor burden and in patients with necrotic hepatic tumors (Figure 5). We cannot currently establish whether the bulk of cells were passively shed form the tumor environment or actively extravasated, but the combined observations could point towards the former. The release of cells is likely facilitated by angiogenesis and unstable vasculature of mCRC and may be affected by other physical properties of the tumor microenvironment. The possibility thus exists that the bulk of cells sampled in this study, perhaps caused by ‘leaky’ tumors, could have been masking even rarer cell populations. The metacluster analysis (Figure 4C and Table 2) also displayed more or less prevalent subgroups of cells of different characteristics that will be further deconvoluted by the ongoing genomic and protein profiling.

The ratio of 35% HD-CTC positive patients in this cohort was comparable to detection rates of CTCs in patients with widespread mCRC using CellSearch® [26–28]. The ʽno cell left behind’ HD-SCA approach usually achieves a higher sensitivity than enrichment-based platforms like CellSearch®. The ’low’ detection rate was in this case expected since patients deemed eligible for surgery have a significantly better prognosis compared to those presenting with unresectable disease at diagnosis [29]. Patients included in this study had generally responded well to neoadjuvant treatment, which in many cases is a requirement for curative intent surgery, with frequently observed shrinkage and necrosis of the metastatic lesions. We currently do not know whether the observed necrosis is a result of neoadjuvant treatment or rapid tumor growth outpacing blood supply. Cell shedding could potentially also be a result of neoadjuvant treatment. However, no treatment had been given for at least 21 days prior to surgery, and the fraction of patients receiving neoadjuvant therapy in the HD-CTC negative group was similar to the positive patients (64 vs 68%). Elevated levels of HD-CTCs in patients with post-treatment necrosis could indicate that presence of HD-CTCs is not necessarily a marker of poor prognosis in this mCRC treatment setting. Long-term (up to 2 years) follow-up samples from these patients are currently being collected to evaluate the prognostic value.

In conclusion, we demonstrate that HD-SCA imaging of single cells from liquid and solid biopsies from multiple mCRC patients reaches the analytical resolution required to quantitatively describe single cell pleomorphism within tumor samples of individual patients and across cohorts. To this end, we established a new approach within the validated HD-SCA platform, introducing quantification of liquid and solid biopsy cell concordance. While the study was limited by the current lack of genomic and/or multiplex proteomic single-cell data, the morphometric characterization of >1000 single cells supports that liquid biopsy cells are related to solid tumor cells in mCRC, and that subgroups of cells with characteristic morphology are present across patients and the liquid and solid cell compartments. The association with tumor presence shows that HD-CTCs have the potential to be used as markers for disease and to monitor disease burden over time. Analysis of tumor touch preparations in parallel with the conventional liquid biopsy cell profiling within the HD-SCA platform can be readily introduced to the workflow to assess the level of concordance within a patient, and to determine the applicability of liquid biopsies for tumor characterization for each individual case. In line with the recently clinically approved AR-V7 predictive test for prostate cancer, we foresee that our approach could pave the way for developing additional CTC assays measuring a condensed set of features of liquid biopsy cells for addressing clinical decision-making.

## METHODS

### Clinical study overview

Patients >18 years of age, diagnosed with surgically resectable hepatic mCRC were enrolled on harmonized, IRB approved clinical protocols at Scripps MDAnderson Cancer Center (La Jolla, CA, USA), USC Norris Comprehensive Cancer Center/Keck School of Medicine (Los Angeles, CA) and Baylor College of Medicine (Houston, TX, USA). Systemic chemotherapy was not administered for at least 21 days prior to resection. Samples were collected at the day of surgery, except for patient CRC712 where the pre-surgery sample was collected 1 week prior. Study data were managed using REDCap. Venous peripheral blood was collected in Cell-free DNA Blood Collection Tubes® (Streck, La Vista, NE, USA) prior to surgery (before anesthesia) and post-surgery. Five touch prep slides were collected from each piece of resected tumor tissue immediately after resection, by gently touching the tissue against standard microscope glass slides [30]. At the time of data analysis, pre-surgery liquid biopsies and a minimum of 1 hepatic touch prep had been collected from 43 patients (Table 1). Post-surgery blood samples were available for 40 of the patients and included in pre- to post-surgery comparison of High Definition-CTCs (HD-CTCs) levels. Ten patients were included in the morphometric characterization for liquid and solid biopsy correlation, based on inclusion criteria described below. Single cell morphometric data from a previously reported study [7] of prostate cancer (PrC) patient PrC33164 was used for comparison.

### HD-SCA workflow and CRC assay development and validation

Matching blood and tumor touch prep samples were processed in parallel within 24 hours after collection as previously described for peripheral blood [9], with nucleated cells plated in a monolayer of 3*10^6^ cells/slide (Marienfeld, Lauda, Germany) before cryobanking. Slides were stained in batches of 50, using the validated, standard HD-SCA protocol with a cocktail of pan-cytokeratin (CK), CK-19, CD45 antibodies and DAPI [9, 31] complemented with a CDX2 monoclonal antibody EPR2764Y (Abcam, Cambridge, UK), and imaged in 2305 frames per slide using automated high-throughput fluorescence scanning microscopy at 10X magnification with exposures and gain set to yield the same background intensity level for normalization purposes. For each slide, a report with 4-channel images and preliminary prediction classifications of candidate cells was reviewed and signed off by an analyst (ASG). Enumeration of cells was converted to concentration based on the sample leukocyte concentration measured at processing, and the number of DAPI positive nuclei detected. Cells of interest were re-imaged at 40X magnification for collection of high-resolution single cell imaging data.

The CRC assay was developed using primary (SW480) and metastatic (LoVo) CRC cell lines and a hepatocellular carcinoma cell line (HepG2) as positive and negative controls, respectively, spiked in whole blood from normal blood donors. Two rabbit monoclonal CDX2 antibody clones (SP54 and EPR2764Y) were evaluated and confirmed to have no cross-reactivity with the standard HD-SCA reagents. The preferred antibody based on signal-to-noise ratio (EPR2764Y) was titrated in 6 dilutions combined with 2 dilutions of the secondary Alexa Fluor 488 goat anti-rabbit antibody (Life Technologies, Waltham, MA, USA) in 3 independent experiments and used at 100X dilution with 500X dilution of secondary goat anti-rabbit Alexa488 in the final assay Non-CRC cells (PANC-1, PC-2, Saos-2), endothelial cells (HUVEC) and white blood cells (WBCs) from normal blood donors showed no CDX2 expression when stained with the assay. The analytical performance in CRC was demonstrated by ability and reproducibility in identifying spiked CRC cells and by comparing intensity levels in model CRC cells and patient HD-CTCs.

### Liquid and solid biopsy cell correlation analysis

The criteria for including a set of matched liquid and solid biopsy samples were based on a preliminary power analysis on data from the first 5 HD-CTC positive samples in the cohort. A subset of 8 features were measured for all 40X images of liquid and solid biopsy cells by creating nuclear and cytoplasmic masks based on the DAPI and CK images, respectively. The segmentation was performed in ImagePro (MediaCybernetics, Rockville, MD, USA). Cohen’s effect sizes were calculated for each feature and patient using the *effsize* package in R (Supplementary Table 1). Mean effect sizes were used to estimate the number of cells needed in each group to reach adequate statistical power (0.8), using the *pwr.t.test* two-sample function in the *pwr* package in R. The number of cells required to reach power 0.8 ranged from 8-20 (Supplementary Table 1), thus it was determined that liquid biopsies should have a minimum of 20 HD-CTCs on the first 2 scanned slides to be included. For the touch prep slides, which frequently had an abundance of cells, the main challenge was identifying clusters of true monolayers of cells that could be reliably segmented. For representative sampling of the tumor, it was decided that slides with >4 monolayer clusters of intact tumor cells should be included. Based on these criteria, 10 patients were included in the correlation analysis. HD-CTCs were relocated and re-imaged at 40X magnification using identical optical setup and exposure times for liquid and solid biopsy cells. Eighty-two features were computed for each cell that could be reliably segmented on the touch preps slides and all HD-CTCs detected on 2 liquid biopsy slides.

All data analyses were conducted in R and displayed using the *ggplot2* package. Boxplots were created from raw measurements of individual features, using the *geom_boxplot* and *geom_jitter* functions. The *caret* package was applied for preprocessing, using the *BoxCox*, *zv*, *center* and *scale* functions on a per patient basis, and the *pca* function in the *FactoMineR* package for PCA. Hierarchical clustering and heatmaps were displayed using the *heatmap.2* function. Dendrograms were analyzed by third level branching to define clusters of cells within each patient. Mean values of features were used for each cluster as input to a metacluster analysis to pinpoint cell groups of different characteristics. Main “clusters of clusters” were defined by second level branching. Spearman correlation of features were computed using the *cor* function and visualized using the *corrplot* package. For correlation analysis of liquid and solid biopsy cells within each patient, the raw measurements were scaled to a 0-1 range. Correlation of liquid and solid biopsy cells was assessed by Pearson correlation coefficients of the profiles of (average) scaled values within each patient. Intraclass Correlation Coefficents (ICC) were calculated using the *ICC* package. Individual p-values for intra-patient liquid versus solid biopsy cells as well as HD-CTCs from CRC versus PrC were calculated using two-tailed, heteroscedastic Student’s t-tests, and pre- and post-surgery samples were compared using 2-sided paired t-test.

## SUPPLEMENTARY MATERIALS AND FIGURES





## Abbreviations

AR: Androgen Receptor
AR-V7: nuclear Androgen Receptor splice variant 7
CAP: College of American Pathologists
CDX2: Caudal type homeobox 2
CK: Cytokeratin
CLIA: Clinical Laboratory Improvement Amendments
CRC: Colorectal Cancer
CTC: Circulating Tumor Cell
CTCC: Circulating Tumor Cell Cluster
ctDNA: circulating-tumor DNA
FFPE: Formalin-Fixed Paraffin-Embedded
HD-CTC: High-Definition Circulating Tumor Cell
HD-SCA: High-Definition Single Cell Analysis
ICC: Intraclass Correlation Coefficient
mCRC: metastatic Colorectal Cancer
N/C: Nuclear-Cytoplasm ratio
PCA: Principal Component Analysis
PrC: Prostate Cancer
RFU: Relative Fluorescence Unit
SDOM: Standard Deviation Over the Mean
WBC: White Blood Cell.
