# Supplementary material for: Single cell correlation analysis of liquid and solid biopsies in metastatic colorectal cancer
Source: Oncotarget. 2019 Dec 17;10(66):7016–30. doi: 10.18632/oncotarget.27271 (PMC6925029; doi:10.18632/oncotarget.27271)
Supplement: Supplementary file 1 [file oncotarget-10-7016-s001.pdf]

Single cell correlation analysis of liquid and solid biopsies in metastatic colorectal cancer

SUPPLEMENTARY MATERIALS

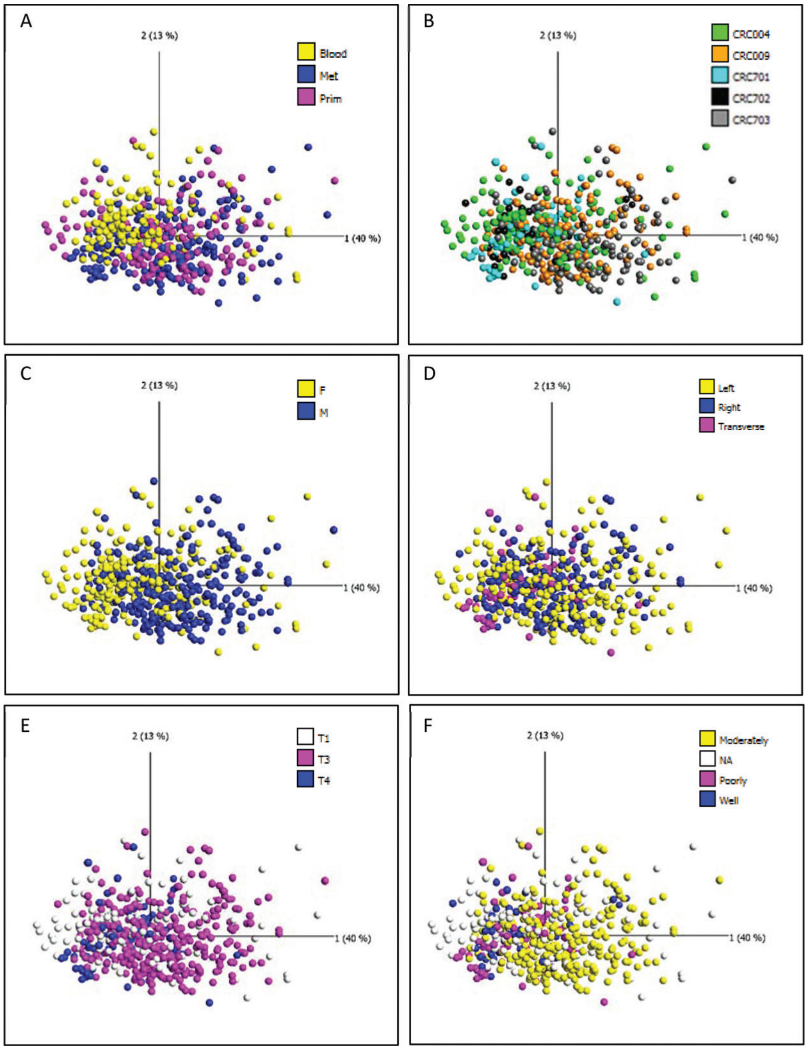

Supplementary Figure 1: Principal component analysis with samples from the first five patients analyzed (974 single cells). Cells are colored by **(A)** tissue type, **(B)** patient, **(C)** gender, **(D)** colon location of primary tumor, **(E)** primary tumor **stage, (F)** primary tumor differentiation.

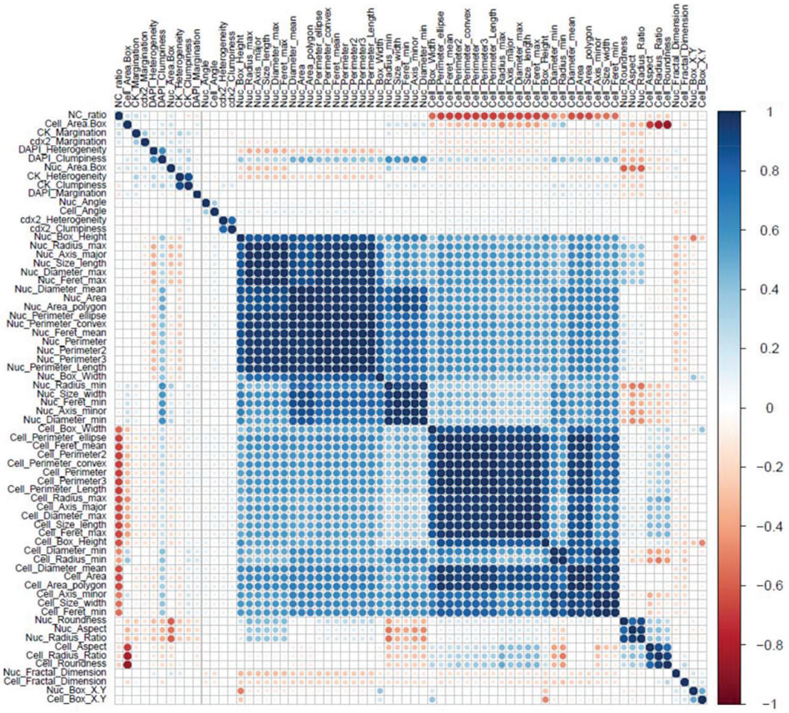

Supplementary Figure 2: Spearman correlation based on data from the complete set of liquid and solid biopsy cells from ten CRC patients. The variables are ordered by hieararchial clustering. The color bar ranges from blue (positive correlation) to red (negative correlation).

Supplementary Table 1: Effect sizes for liquid and solid biopsy single cell comparison.

	**CRC-SC004**	**CRC-SC009**		**CRC-USC701**	**CRC-USC702**	**CRC-USC703**		
	CTCs vs met.	CTCs vs met.	CTCs vs prim.	CTCs vs met.	CTCs vs met.	CTCs vs met.	CTCs vs prim.	
No. of cells measured	*62 vs 38*	*25 vs 30*	*25 vs 28*	*29 vs26*	*15 vs 19*	*24 vs 48*	*24 vs 49*	Mean effect size
Nuclear Area	0.47	1.66	0.13	0.55	0.34	1.96	1.60	0.96
DAPI Den./Inten. (mean)	1.33	2.01	0.71	3.22	2.49	1.59	1.50	1.84
Nuclear Roundness	0.35	1.47	1.89	2.00	0.35	0.93	0.34	1.05
Cell Area	0.17	1.46	1.03	0.60	0.72	1.01	0.57	0.79
CK Den./ Inten. (mean)	3.01	2.04	2.87	2.96	3.5	3.8	2.57	2.96
Cell Roundness	0.38	0.17	0.22	0.07	0.18	0.11	0.09	0.17
CDX2 Den./Inten. (mean)	1.50	1.20	4.19	1.18	0.65	0.60	0.13	1.35
N/C-ratio	0.43	0.54	0.87	0.78	0.10	0.61	0.57	0.56
Mean effect size	0.96	1.32	1.49	1.42	1.04	1.33	0.92	1.21

Effect sizes computed for a subset of 8 features. Values are colored according to effect size, from white to dark blue for trivial, small, moderate and large difference effects**.**

Supplementary Table 2: Intra-patient solid and liquid biopsy cell comparison.

See Supplementary Table 2: Red=higher and green=lower in solid versus liquid biopsy cells ($p<0.01$). Only features with significant differences in one or more patients are listed
