# Supplementary material for: Single cell correlation analysis of liquid and solid biopsies in metastatic colorectal cancer
Source: Oncotarget. 2019 Dec 17;10(66):7016–30. doi: 10.18632/oncotarget.27271 (PMC6925029; doi:10.18632/oncotarget.27271)
Supplement: Supplementary file 2 [file oncotarget-10-7016-s002.docx]

**Supplementary Table 2:** Intra-patient solid and liquid biopsy cell comparison.
Red=higher and green=lower in solid versus liquid biopsy cells (p<0.01). Only features with significant differences in one or more patients are listed.

|  | BCM119 | BCM120 | SC004 | SC009 | USC701 | USC702 | USC703 | USC709 | USC711 | USC712 |
| --- | --- | --- | --- | --- | --- | --- | --- | --- | --- | --- |
| NC_ratio |  |  |  |  |  |  |  |  |  |  |
| Nuc_Area |  |  |  |  |  |  |  |  |  |  |
| Nuc_Aspect |  |  |  |  |  |  |  |  |  |  |
| Nuc_Area/Box |  |  |  |  |  |  |  |  |  |  |
| Nuc_Box_X/Y |  |  |  |  |  |  |  |  |  |  |
| Nuc_Axis_major |  |  |  |  |  |  |  |  |  |  |
| Nuc_Axis_minor |  |  |  |  |  |  |  |  |  |  |
| Nuc_Diameter_max |  |  |  |  |  |  |  |  |  |  |
| Nuc_Diameter_min |  |  |  |  |  |  |  |  |  |  |
| Nuc_Diameter_mean |  |  |  |  |  |  |  |  |  |  |
| Nuc_Radius_max |  |  |  |  |  |  |  |  |  |  |
| Nuc_Radius_min |  |  |  |  |  |  |  |  |  |  |
| Nuc_Perimeter |  |  |  |  |  |  |  |  |  |  |
| Nuc_Radius_Ratio |  |  |  |  |  |  |  |  |  |  |
| Nuc_Roundness |  |  |  |  |  |  |  |  |  |  |
| Nuc_Size_length |  |  |  |  |  |  |  |  |  |  |
| Nuc_Size_width |  |  |  |  |  |  |  |  |  |  |
| Nuc_Perimeter2 |  |  |  |  |  |  |  |  |  |  |
| Nuc_Perimeter_convex |  |  |  |  |  |  |  |  |  |  |
| Nuc_Perimeter_ellipse |  |  |  |  |  |  |  |  |  |  |
| Nuc_Area_polygon |  |  |  |  |  |  |  |  |  |  |
| Nuc_Fractal_Dimension |  |  |  |  |  |  |  |  |  |  |
| Nuc_Box_Width |  |  |  |  |  |  |  |  |  |  |
| Nuc_Box_Height |  |  |  |  |  |  |  |  |  |  |
| Nuc_Feret_min |  |  |  |  |  |  |  |  |  |  |
| Nuc_Feret_max |  |  |  |  |  |  |  |  |  |  |
| Nuc_Feret_mean |  |  |  |  |  |  |  |  |  |  |
| DAPI_Margination |  |  |  |  |  |  |  |  |  |  |
| DAPI_Heterogeneity |  |  |  |  |  |  |  |  |  |  |
| DAPI_Clumpiness |  |  |  |  |  |  |  |  |  |  |
| Nuc_Perimeter3 |  |  |  |  |  |  |  |  |  |  |
| Nuc_Perimeter_Length |  |  |  |  |  |  |  |  |  |  |
| Cell_Area |  |  |  |  |  |  |  |  |  |  |
| Cell_Box_X/Y |  |  |  |  |  |  |  |  |  |  |
| Cell_Axis_major |  |  |  |  |  |  |  |  |  |  |
| Cell_Axis_minor |  |  |  |  |  |  |  |  |  |  |
| Cell_Diameter_max |  |  |  |  |  |  |  |  |  |  |
| Cell_Diameter_min |  |  |  |  |  |  |  |  |  |  |
| Cell_Diameter_mean |  |  |  |  |  |  |  |  |  |  |
| Cell_Radius_max |  |  |  |  |  |  |  |  |  |  |
| Cell_Radius_min |  |  |  |  |  |  |  |  |  |  |
| Cell_Perimeter |  |  |  |  |  |  |  |  |  |  |
| Cell_Roundness |  |  |  |  |  |  |  |  |  |  |
| Cell_Size_length |  |  |  |  |  |  |  |  |  |  |
| Cell_Size_width |  |  |  |  |  |  |  |  |  |  |
| Cell_Perimeter2 |  |  |  |  |  |  |  |  |  |  |
| Cell_Perimeter_convex |  |  |  |  |  |  |  |  |  |  |
| Cell_Perimeter_ellipse |  |  |  |  |  |  |  |  |  |  |
| Cell_Area_polygon |  |  |  |  |  |  |  |  |  |  |
| Cell_Fractal_Dimension |  |  |  |  |  |  |  |  |  |  |
| Cell_Box_Width |  |  |  |  |  |  |  |  |  |  |
| Cell_Box_Height |  |  |  |  |  |  |  |  |  |  |
| Cell_Feret_min |  |  |  |  |  |  |  |  |  |  |
| Cell_Feret_max |  |  |  |  |  |  |  |  |  |  |
| Cell_Feret_mean |  |  |  |  |  |  |  |  |  |  |
| CK_Margination |  |  |  |  |  |  |  |  |  |  |
| CK_Heterogeneity |  |  |  |  |  |  |  |  |  |  |
| CK_Clumpiness |  |  |  |  |  |  |  |  |  |  |
| Cell_Perimeter3 |  |  |  |  |  |  |  |  |  |  |
| Cell_Perimeter_Length |  |  |  |  |  |  |  |  |  |  |
| cdx2_Heterogeneity |  |  |  |  |  |  |  |  |  |  |
| cdx2_Clumpiness |  |  |  |  |  |  |  |  |  |  |
| cdx2_Margination |  |  |  |  |  |  |  |  |  |  |
